# The Melatonin Receptor Agonist Ramelteon Induces Cardioprotection that Requires MT2 Receptor Activation and Release of Reactive Oxygen Species

**DOI:** 10.1007/s10557-020-06972-4

**Published:** 2020-03-31

**Authors:** Martin Stroethoff, Lukas Goetze, Carolin Torregroza, Sebastian Bunte, Annika Raupach, André Heinen, Alexander Mathes, Markus W. Hollmann, Ragnar Huhn

**Affiliations:** 1 Department of Anesthesiology, University Hospital Duesseldorf, Moorenstr. 5, 40225 Duesseldorf, Germany; 2 Institute of Cardiovascular Physiology, Heinrich-Heine-University Duesseldorf, Universitaetsstr. 1, 40225 Duesseldorf, Germany; 3 Department of Anesthesiology and Intensive Care Medicine, University Hospital Cologne, Kerpener Str. 62, 50937 Cologne, Germany; 4 Department of Anesthesiology, Amsterdam University Medical Center (AUMC), Location AMC, Meiberdreef 9, 1100 DD Amsterdam, Netherlands

**Keywords:** Cardioprotection, Pharmacology, Melatonin receptor agonist

## Abstract

**Purpose:**

The melatonin receptor (MT) agonist ramelteon has a higher affinity to MT1 than for MT2 receptors and induces cardioprotection by involvement of mitochondrial potassium channels. Activation of mitochondrial potassium channels leads to release of free radicals. We investigated whether (1) ramelteon-induced cardioprotection is MT2 receptor specific and (2) if free radicals are involved in ramelteon-induced cardioprotection.

**Methods:**

Hearts of male Wistar rats were randomized, placed on a Langendorff system, and perfused with Krebs-Henseleit buffer at a constant pressure of 80 mmHg. All hearts were subjected to 33 min of global ischemia and 60 min of reperfusion. Before ischemia hearts were perfused with ramelteon (Ram) with or without the MT2 receptor inhibitor 4-phenyl-2-propionamidotetralin (4P-PDOT+Ram, 4P-PDOT). In subsequent experiments, ramelteon was administered together with the radical oxygen species (ROS) scavenger N-2-mercaptopropionylglycine (MPG+Ram). To determine whether the blockade of ramelteon-induced cardioprotection can be restored, we combined ramelteon and MPG with mitochondrial permeability transition pore (mPTP) inhibitor cyclosporine A (CsA) at different time points. Infarct size was determined by triphenyltetrazolium chloride (TTC) staining.

**Results:**

Ramelteon-induced infarct size reduction was completely blocked by 4P-PDOT and MPG. Ramelteon and MPG combined with CsA before ischemia were not cardioprotective but CsA at the onset of reperfusion could restore infarct size reduction.

**Conclusions:**

This study shows for the first time that despite the higher affinity to MT1 receptors, (1) ramelteon-induced cardioprotection involves MT2 receptors, (2) cardioprotection requires ROS release, and (3) inhibition of the mPTP can restore infarct size reduction.

## Introduction

Reperfusion after myocardial ischemia is essential for the survival of the patient. Unfortunately, reperfusion itself causes myocardial injury [1] thus being contraproductive in such situations. Therefore, cardioprotective strategies that could counteract this situation and limit myocardial injury would be of great value.

Preconditioning is a strong cardioprotective intervention to reduce the infarct size after ischemia-reperfusion injury [2]. Cardioprotection can be induced by ischemic preconditioning with short cycles of ischemia and reperfusion or realized by pharmacologic preconditioning with drugs like volatile anesthetics [3], opioids [4], or noble gases [5]. Previously, we showed that administration of the melatonin receptor (MT) agonist ramelteon prior to ischemia is cardioprotective in a concentration-dependent manner [6]. Three subtypes of MT receptors are known, whereas ramelteon has a higher affinity to MT1 than to MT2 receptors and less affinity to MT3 receptors [7]. Own previous data showed that the non-selective MT receptor antagonist luzindole completely abolished ramelteon-induced infarct size reduction [6]. Because luzindole is non-selective for a MT receptor subtype, it remains unclear whether the higher affinity to MT1 and thus the activation of MT1 receptors is essentially responsible for the cardioprotection by ramelteon.

Recently, we demonstrated that the cardioprotective effect of ramelteon is mediated by activation of mitochondrial potassium channels, e.g., mitochondrial ATP-sensitive potassium channels (mK_ATP_-channels) and mitochondrial calcium-sensitive potassium channels (mK_Ca_-channels) [6]. The blockade of these channels completely abrogated the cardioprotection by ramelteon [6]. Activation of mitochondrial potassium channels leads to the release of reactive oxygen species (ROS) that are involved in the signal transduction cascade of preconditioning by inhibiting the opening of the mitochondrial permeability transition pore (mPTP) [8]. Until now, it is unknown, if the release of ROS plays also a pivotal role in the context of ramelteon-induced cardioprotection.

Therefore, we set out to determine if (1) ramelteon, despite its higher affinity to MT1 receptors, also requires activation of MT2 receptors for inducing a cardioprotective effect, if (2) the release of ROS is essential in ramelteon-induced infarct size reduction, and if (3) inhibition of mPTP with cyclosporine A (CsA) restores abolished ramelteon-induced cardioprotection by scavenging of ROS.

## Methods

This investigation was in accordance with the Guide for the Care and Use of Laboratory Animals published by the US National Institutes of Health (NIH Publication No. 85–23, revised 1996) and was approved from the Animal Ethics Committee of the University of Düsseldorf, Germany (O27/12). Experiments were done and results were reported in accordance with the ARRIVE guidelines. The animals are provided and housed by the Central Facility for Animal Research, University of Duesseldorf, Germany.

### Surgical Preparation

The surgical preparation was performed as described previously [9, 10]. In brief, male Wistar rats (mean body weight part 1: 297 ± 15 g, part 2: 273 ± 12 g) were anesthetized by intraperitoneal injection of 90 mg/kg pentobarbital and decapitated. After thoracotomy, the hearts were excised and mounted on a Langendorff system and were perfused at a constant pressure of 80 mmHg with a Krebs-Henseleit solution (pH 7.38–7.43), enriched with 95% O_2_ and 5% CO_2_. The solution contains (in mM) the following: 118 NaCl, 4.7 KCl, 1.2 MgSO_4_, 1.17 KH_2_PO_4_, 24.9 NaHCO_3_, 2.52 CaCl_2_, 0.5 EDTA, 11 glucose, and 1 lactate at 37 °C. A fluid-filled balloon was inserted into the left ventricle and the end-diastolic pressure was set at 2–8 mmHg. Heart rate, the left ventricular end-systolic pressure (LVESP), the left ventricular end-diastolic pressure (LVEDP), and coronary flow were measured continuously and digitized at a sampling rate of 500 Hz by use of an analogue to digital converter system (PowerLab/8SP, ADInstruments Pty Ltd., Castle Hill, Australia). The left ventricular developed pressure (phasic LVP) was calculated as LVESP–LVEDP. Data were continuously recorded on a personal computer using Chart for Windows v5.0 (ADInstruments Pty Ltd).

### Experimental Protocol

**Fig. 1:**
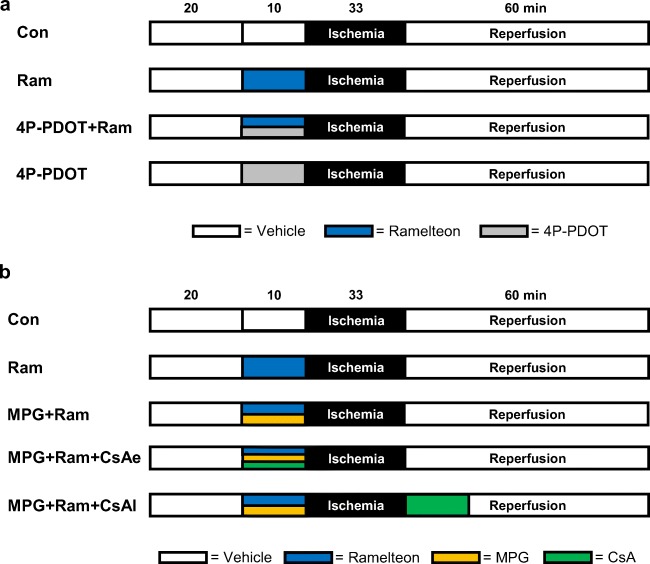
**a**, **b** Experimental protocol. Con, control; Ram, ramelteon; 4P-PDOT, 4-phenyl-2-propionamidotetralin; MPG, N-2-mercaptopropionylglycine; CsA, cyclosporine A

The study was divided in two parts. In the first part, we investigated whether ramelteon-induced cardioprotection involves activation of MT2 receptors. Previously, we demonstrated that the lowest cardioprotective concentration of ramelteon is 0.03 μM [6]. The selective MT2 receptor antagonist 4-phenyl-2-propionamidotetralin (4P-PDOT) was administered in a concentration of 1 μM [11, 12]. 4P-PDOT and ramelteon were administered over a time period of 10 min respectively before global ischemia and reperfusion (Fig. 1). Each group underwent a baseline period of 20 min, 33 min of global ischemia, and 60 min of reperfusion, respectively. Global ischemia of 33 min caused a sufficient infarct size, representing the primary endpoint of this study, to show relevant cardioprotective effects. Additionally, this degree of myocardial damage does not completely impair myocardial function during reperfusion. In all groups, global myocardial ischemia was induced by stopping the perfusion of the whole heart. Rat hearts were randomly assigned into four groups (*n* = 6–7 per group):

Control (Con, *n* = 6): hearts were perfused with Krebs-Henseleit solution for 10 min.

Ramelteon (Ram, *n* = 6): hearts were perfused with 0.03 μM ramelteon for 10 min.

4-Phenyl-2-propionamidotetralin+ramelteon (4P-PDOT+Ram, *n* = 6): hearts were perfused with 1 μM 4P-PDOT [11, 12] combined with 0.03 μM ramelteon for 10 min.

4-Phenyl-2-propionamidotetralin (4P-PDOT, *n* = 7): to rule out an effect on myocardial infarction size by 4P-PDOT itself, 4P-PDOT (1 μM) was also administered for 10 min without ramelteon.

The second part was designed to investigate whether cardioprotection by ramelteon involves free oxygen radicals. Rat hearts were randomly assigned to five experimental groups (*n* = 6–8 per group, Fig. 1b):

Control (Con, *n* = 8): hearts were perfused with Krebs-Henseleit solution for 10 min.

Ramelteon (Ram, *n* = 7): hearts were perfused with 0.03 μM ramelteon for 10 min.

N-2-Mercaptopropionylglycine+ramelteon (MPG+Ram, *n* = 6): hearts were perfused with ramelteon in combination with the ROS scavenger MPG (1 mM) for 10 min [13]. From own data, we know that MPG had no effect on infarct size [13].

N-2-Mercaptopropionylglycine+ramelteon+cyclosporine A early (MPG+Ram+CsAe, *n* = 7): to investigate if cardioprotection can be restored, MPG and ramelteon were combined with the mPTP inhibitor CsA (0.2 μM) [14] for 10 min before global ischemia. Previously, we demonstrated in an comparable setup that CsA alone significantly reduced infarct size [14].

N-2-Mercaptopropionylglycine+ramelteon+cyclosporine A late (MPG+Ram+CsAl, *n* = 6): to investigate if cardioprotection can be restored, MPG and ramelteon were combined with CsA (0.2 μM) [14] for 10 min after global ischemia.

After reperfusion, the hearts were cut into transverse slices, starting from the cardiac apex to just before the cardiac valvular plane. The slices were stained with 0.75% triphenyltetrazolium chloride (TTC) solution. The size of the infarcted area was determined by planimetry using the SigmaScan Pro 5® computer software (SPSS Science Software, Chicago, IL).

### Statistical Analysis

#### Sample Size Analysis

The calculated sample size was *n* = 6–7 for detecting a 25% mean difference in infarct size (power 80%, *α* < 0.05 (two-tailed)).

#### Statistical Approach

All data are expressed as mean ± SD. Analysis of statistical data was performed using GraphPad StatMate TM (GraphPad Software, San Diego, CA, USA). Part 1 and part 2 of the study were analyzed separately. A researcher blinded to the experimental groups evaluated the infarct sizes. The infarct sizes were analyzed by one-way analysis of variance (ANOVA) followed by Tukey’s post hoc test. Comparisons of hemodynamic data among groups or among time points in a group were analyzed by two-way ANOVA followed by Tukey’s post hoc test for group effects and Dunnett’s post hoc test for time effects. Changes were considered statistically significant if *P* values were less than 0.05.

## Results

### Infarct Size

**Fig. 2:**
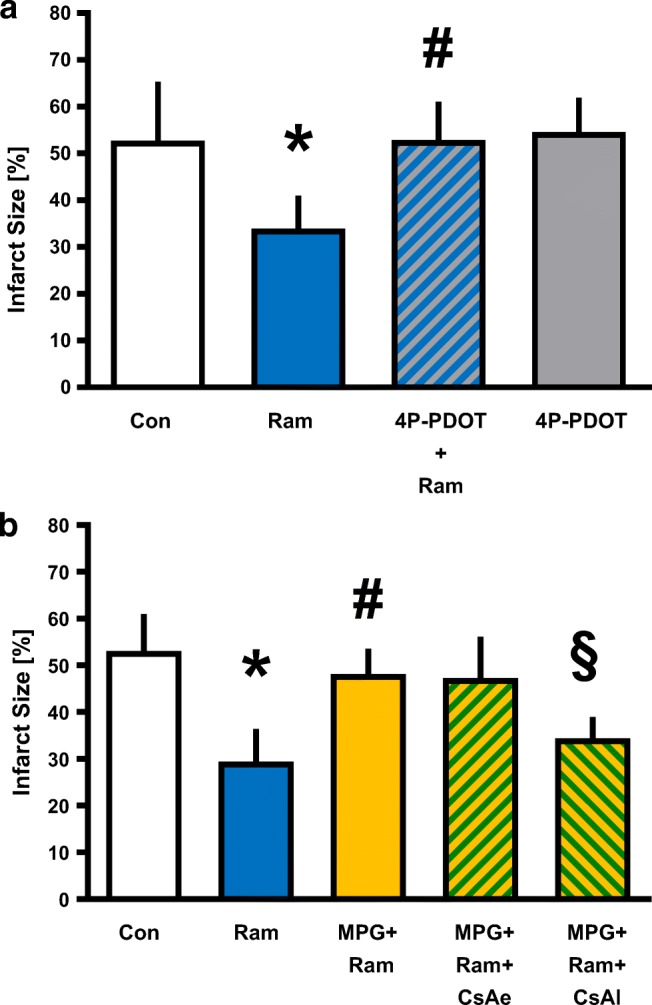
Infarct size measurement. **a** Histogram shows the infarct size of controls, ramelteon, and 4P-PDOT—with and without ramelteon. **b** Histogram shows the infarct size of controls, ramelteon, MPG, and CsA administered at two different time points. Data are presented as means ± SD, *P < 0.05 vs. Con, ^#^*P* < 0.05 vs. Ram, ^§^*P* < 0.05 vs. MPG+Ram. Con, control; Ram, ramelteon; 4P-PDOT, 4-phenyl-2-propionamidotetralin; MPG, N-2-mercaptopropionylglycine; CsA, cyclosporine A

In part 1 of the study, the infarct size of the control group (Con) was 53 ± 13% (Fig. 2). Ramelteon (Ram) at 0.03 μM reduced the infarct size to 34 ± 7% (*P* = 0.0082 vs. Con). The MT2 receptor inhibitor 4P-PDOT completely abolished ramelteon-induced cardioprotection (4P-PDOT+Ram 53 ± 8%; *P* = 0.0074 vs. Ram,) without having an effect on the infarct size itself (4P-PDOT: 55 ± 7%; *P* = 0.9821 vs. Con).

Unfortunately, there is no commercial MT1 receptor inhibitor available. In a previous study, we showed that the unspecific MT receptor inhibitor luzindole completely abolished the cardioprotective effect of ramelteon [6].

In part 2 of the study, ramelteon reduced significantly the infarct size compared with the control group (Con 53 ± 8%; Ram 29 ± 7%; *P* < 0.0001 vs. Con, Fig. 2b). The ROS scavenger MPG completely abolished the infarct size reduction of ramelteon (MPG+Ram 48 ± 5%; *P* = 0.0004 vs. Ram,) indicating that free oxygen radicals are critically involved in ramelteon-induced cardioprotection. Combination of MPG and ramelteon with the mPTP inhibitor CsA at the same time before global ischemia had no effect on infarct size (MPG+Ram+CsAe 47 ± 9%; *P* = 0.5328 vs. Con). In the literature, a hint for an interaction between ramelteon and CsA is described [15]. To bypass a simultaneous application of both substances, we determined if CsA administration at the onset of reperfusion could restore the infarct size-reducing effect. Our results show that subsequent administration of CsA completely restored the cardioprotective effect of ramelteon (MPG+Ram+CsAl 34 ± 5%; *P* = 0.0161 vs. MPG+Ram).

### Cardiac Function

Table 1 and Table 2 show hemodynamic variables of both parts of the study. In part 1, there were no differences in heart rate between the study groups (Table 1). The left ventricular developed pressure (phasic LVP) and coronary flow were significantly lower during reperfusion in all groups compared with baseline (Table 1), with no differences between groups. In part 2 of the study, heart rate was significantly lower in the control group at the end of the experiments compared with baseline (Table 2). During reperfusion, phasic LVP and coronary flow were decreased (all *P* < 0.05 vs. baseline, Table 2). The MPG+Ram and the MPG+Ram+CsAl groups showed significant differences before ischemia compared with Con (Table 2).

**Table 1:** Hemodynamic variables in part 1

	Baseline	Pretreatment	Reperfusion	
			30	60
Heart rate (bpm)				
Con	341 ± 51	333 ± 50	281 ± 54	255 ± 75
Ram	311 ± 32	304 ± 28	257 ± 44	251 ± 33
4P-PDOT+Ram	296 ± 39	276 ± 10	213 ± 55	247 ± 32
4P-PDOT	301 ± 32	270 ± 22	211 ± 71	220 ± 44
Phasic LVP (mmHg)				
Con	134 ± 7	146 ± 6	24 ± 13*	30 ± 11*
Ram	130 ± 19	136 ± 18	18 ± 12*	29 ± 15*
4P-PDOT+Ram	146 ± 23	153 ± 24	23 ± 10*	30 ± 10*
4P-PDOT	134 ± 21	148 ± 19	35 ± 18*	40 ± 11*
Coronary flow (ml/min)				
Con	15 ± 3	15 ± 4	8 ± 2*	7 ± 2*
Ram	15 ± 2	15 ± 2	7 ± 1*	6 ± 1*
4P-PDOT+Ram	15 ± 3	13 ± 3	6 ± 1*	5 ± 1*
4P-PDOT	14 ± 3	14 ± 3	7 ± 2*	6 ± 2*

*Con*, control; *Ram*, ramelteon; *4P-PDOT*, 4-phenyl-2-propionamidotetralin; *LVP*, left ventricular pressure

*P < 0.05 vs. baseline

^#^*P* < 0.05 vs. Con

**Table 2:** Hemodynamic variables in part 2

	Baseline	Pretreatment	Reperfusion	
			30	60
Heart rate (bpm)				
Con	291 ± 27	278 ± 18	205 ± 56	181 ± 55*
Ram	287 ± 30	266 ± 32	239 ± 46	215 ± 45
MPG+Ram	299 ± 32	301 ± 59	229 ± 74	236 ± 34
MPG+Ram+CsAe	296 ± 26	278 ± 24	227 ± 57	216 ± 34
MPG+Ram+CsAl	273 ± 20	248 ± 26	224 ± 53	181 ± 25
Phasic LVP (mmHg)				
Con	112 ± 24	108 ± 30	15 ± 8*	28 ± 9*
Ram	119 ± 33	113 ± 38	13 ± 4*	19 ± 5*
MPG+Ram	139 ± 18^#^	137 ± 14^#^	10 ± 5*	17 ± 7*
MPG+Ram+CsAe	120 ± 18	121 ± 26	17 ± 8*	20 ± 12*
MPG+Ram+CsAl	143 ± 13^#^	137 ± 8^#^	20 ± 19*	27 ± 14*
Coronary flow (ml/min)				
Con	13 ± 2	12 ± 2	6 ± 2*	5 ± 2*
Ram	14 ± 2	13 ± 3	6 ± 2*	5 ± 2*
MPG+Ram	16 ± 3	18 ± 2^#^	7 ± 2*	6 ± 2*
MPG+Ram+CsAe	15 ± 2	15 ± 3	6 ± 1*	5 ± 1*
MPG+Ram+CsAl	13 ± 2	14 ± 2	5 ± 1*	4 ± 1*

*Con*, control; *Ram*, ramelteon; *MPG*, N-2-mercaptopropionylglycine; *CsA*, cyclosporine A; *CsAe*, CsA early application; *CsAl*, CsA late application; *LVP*, left ventricular pressure

*P < 0.05 vs. baseline

^#^*P* < 0.05 vs. Con

## Discussion

The results of the present study, together with own previously published data, show that the cardioprotective effect of the melatonin receptor agonist ramelteon requires activation of MT2 receptor. Furthermore, we demonstrate that the release of reactive oxygen species is critically involved in the signaling cascade of ramelteon-induced cardioprotection.

The melatonin receptor agonist ramelteon is a clinically used medication for treatment of insomnia with specific action on MT receptors without affecting other types of receptors [7]. A cardioprotective effect for melatonin is described in the literature [16], and previously, we showed that ramelteon reduced the myocardial infarct size comparable with melatonin in a concentration-dependent manner [6]. We further reported that the non-selective MT receptor antagonist luzindole completely abolished the infarct size reduction induced by ramelteon [6]. However, ramelteon has an 8 times higher affinity to MT1 than to MT2 receptors [7], suggesting a major role of MT1 receptors in the context of ramelteon-induced cardioprotection. In the present study, the MT2 receptor antagonist 4P-PDOT abrogated the ramelteon-induced infarct size reduction indicating a requirement of the MT2 receptor for cardioprotection. Based on the abovementioned higher affinity to the MT1 receptor and the data from our previous study [6]—complete blockade of infarct size reduction by the non-selective MT receptor antagonist luzindole—we suggest that for the cardioprotective effect of ramelteon, activation of both MT receptors are required.

Activation of mitochondrial potassium channels, e.g., mitochondrial ATP-sensitive potassium (mK_ATP_) channels or mitochondrial calcium-sensitive potassium (mK_Ca_) channels, plays a pivotal role in the signaling cascade of preconditioning. We demonstrated that both channels are involved in ramelteon-induced cardioprotection [6]. There is evidence that activation of mK_Ca_-channels inhibits opening of the mPTP as a key element in the underlying mechanism of myocardial protection [17]. This effect seems to be mediated by regulation of ROS levels as Stowe et al. demonstrated that cardioprotection induced by the mK_Ca_-channel activation depends on ROS that are generated during channel activation. Furthermore, the pharmacological activation of mK_Ca_-channels in isolated heart mitochondria regulates mitochondrial bioenergetics to slightly increase ROS generation [8]. This suggests that a small increase in mitochondrial ROS generation acts as a trigger signal for cardioprotection with the consequence of a strong reduction of the subsequent ROS burst occurring during early reperfusion after ischemia [18]. Our data are in line with the concept of ROS as triggering signal of protection by the mK_Ca_-channel activation as MPG administration during ramelteon pretreatment blocked the cardioprotective effect of this compound. However, the exact role of ROS in different administration protocols of ramelteon, i.e., treatment before and/or after ischemia, remains unclear and should be investigated by measuring ROS release directly.

In addition, it remains unclear which mechanism is responsible for the regulation of mK_Ca_-channels by ramelteon. Previously, we demonstrated that cyclic adenosine-mono-phosphate (cAMP) and protein kinase A (PKA) are upstream regulators of mK_Ca_-channels [19]. Furthermore, it was shown that phospholipase C and guanylylcyclase are regulated by MT2 receptors [20, 21]. If these upstream regulators are influenced by MT2 receptor, the activation has to be elucidated in future studies.

**Fig. 3:**
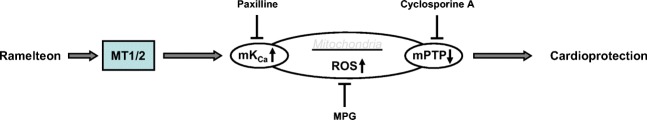
Possible signaling cascade of ramelteon-induced cardioprotection against myocardial ischemia-reperfusion injury in rats. MT, melatonin receptor; mK_Ca_-channel, mitochondrial large-conductance Ca^2+^-sensitive potassium (mK_Ca_) channel; Paxilline, mK_Ca_-channel inhibitor; ROS, radical oxygen species; MPG, N-2-mercaptopropionylglycine, ROS scavenger; mPTP, mitochondrial permeability transition pore; Cyclosporine A, mPTP inhibitor

Opening of the mPTP is associated with cell death and loss of myocardial protection. In the present study, we tried to restore the cardioprotective effect of ramelteon by inhibiting the mPTP with CsA. Interestingly, combined administration of MPG, ramelteon, and CsA before ischemia could not restore cardioprotection. In the literature, there is a hint for an interaction between ramelteon and CsA [15]. As opening of the mPTP occurs at the onset of reperfusion, we added an additional group employing subsequent administration of CsA at the start of reperfusion. This subsequent CsA administration again led to a significant infarct size reduction. Interestingly, we detected a strong infarct size-reducing effect but no hemodynamic improvement during the reperfusion period. Consistent with current literature [22] for isolated perfused hearts, infarct size seems the most sensitive marker to assess cardioprotection. The exact reason for this is unclear, but the occurrence of myocardial stunning is often discussed, especially after global ischemia. Thus, combining the results from the present study together with our previous data [6], we suggest that ramelteon acts via activation of mitochondrial potassium channels resulting in release of ROS and downstream inhibition of the mPTP (Fig. 3). However, we think that CsA does not directly reverse the effect of MPG and that our results rather suggest that the restoration of infarct size reduction is based on inhibition of the mPTP located downstream in the signaling cascade.

In contrast to pre-clinical animal studies, the cardioprotective potential of mPTP inhibition in clinical trials is less clear. Piot et al. showed in a small proof-of-concept trial that administration of CsA before percutaneous coronary intervention lowers creatine kinase release by 40% [23] and triggers a reduction of infarct size by 20% [24]. The CIRCUS trial did, however, not confirm these promising results. The trial failed to show any effect of CsA on a composite endpoint of death, hospitalization for heart failure, and adverse remodeling [25]. However, it is worth mentioning that in the study from Piot et al., patients within < 4 h from symptom onset before reperfusion were recruited, whereas in the CIRCUS trial, the time from symptom onset to reperfusion could be up to 12 h. Therefore, it is questionable whether there is still tissue 12 h after myocardial ischemia that could benefit from a protection and thus the timing of CsA administration is certainly decisive.

The lack of a cardioprotective effect by simultaneous administration of MPG with ramelteon and CsA should be emphasized on a clinical perspective, since cardioprotective properties of ramelteon would be lost when given in combination with specific drugs. Furthermore, also various comorbidities like hyperglycemia/diabetes [26], aging [10], or medications [27] were shown to affect cardioprotection by preconditioning. Previously, hyperglycemia, for instance, blocked the cardioprotective effect of the volatile anesthetic sevoflurane but the infarct size reduction was restored by inhibition of the mPTP with CsA at the onset of reperfusion [28]. Thus, even in the presence of concomitant diseases and/or various medications, abrogated cardioprotection could be restored by mPTP inhibition. Further studies addressing ramelteon-induced cardioprotection in the context of comorbidities are warranted.

It is a limitation of our study that we did not measure ROS production. Therefore, the question how much ROS that is necessary for triggering the cardioprotective effect of ramelteon remains unanswered. Furthermore, we did not study MPG and CsA alone. However, we previously demonstrated in an identical experimental setup that MPG had no effect on infarct size [13] and that CsA alone induced the infarct size reduction [14]. For ethical reasons, we did not repeat these experiments in our current study.

## Conclusion

The results of the present study show that the MT receptor agonist ramelteon requires activation of MT2 receptors for inducing cardioprotection. Furthermore, the cardioprotective effect of ramelteon is completely abolished by ROS blockade but can be restored by subsequent inhibition of the mPTP as a downstream target in the signaling cascade. The cardioprotective properties of ramelteon offer new perspectives and opportunities for this drug. Further studies should elucidate the exact underlying mechanism of ramelteon-induced cardioprotection.
